# Nemaline myopathy and heart failure: role of ivabradine; a case report

**DOI:** 10.1186/1471-2261-15-5

**Published:** 2015-01-19

**Authors:** Filippo M Sarullo, Giuseppe Vitale, Antonino Di Franco, Silvia Sarullo, Ylenia Salerno, Laura Vassallo, Emanuela Petrona Baviera, Stefania Marazia, Giorgio Mandalà, Gaetano A Lanza

**Affiliations:** Cardiovascular Rehabilitation Unit, Buccheri la Ferla Fatebenefratelli Hospital, Via Salvatore Puglisi n. 15, Palermo, Italy; Department of Cardiology, Catholic University of Sacred Heart, Rome, Italy; University of Palermo, Faculty of Medicine, Palermo, Italy; Department of Cardiology, M. Giannuzzi Hospital, Manduria, Taranto Italy

**Keywords:** Nemaline myopathy, Heart failure, Dilated cardiomyopathy, Ivabradine

## Abstract

**Background:**

Nemaline myopathy (NM) is a rare congenital myopathy characterized by muscle weakness, hypotonia and the presence in muscle fibers of inclusions known as nemaline bodies and a wide spectrum of clinical phenotypes, ranging from severe forms with neonatal onset to asymptomatic forms. The adult-onset form is heterogeneous in terms of clinical presentation and disease progression. Cardiac involvement occurs in the minority of cases and little is known about medical management in this subgroup of NM patients. We report a rare case of heart failure (HF) in a patient with adult-onset NM in whom ivabradine proved to be able to dramatically improve the clinical picture.

**Case presentation:**

We report a case of a 37-year-old man with adult-onset NM, presenting with weakness and hypotonia of the proximal limb muscles and shoulder girdle, severely limiting daily activities. He developed progressive HF over a period of 6 months while attending a rehabilitation program, with reduced left ventricular ejection fraction (LVEF = 20%), manifested by dyspnea and signs of systemic congestion. The patient was started HF therapy with enalapril, carvedilol, spironolactone and loop diuretics. Target HF doses of these drugs (including carvedilol) were not reached because of symptomatic hypotension causing a high resting heart rate (HR) ≥70 beats per minute (bpm). Further deterioration of the clinical picture occurred with several life-threatening arrhythmic episodes requiring external defibrillation. An implantable cardioverter defibrillator (ICD) was then implanted. Persistent high resting HR was successfully treated with ivabradine with HR lowering from 90 bpm to 55 bpm at 1 month follow up, LVEF rising to 50% at 3 month follow up and to 54% at 2,5 year follow up. To date no more hospitalizations for heart failure occurred. A single hospitalization due to aspiration pneumonia required insertion of a tracheostomy tube to protect airways from further aspiration. At present, the patient is attending a regular rehabilitation program with net improvement in neuromotor control and less limitations in daily activities.

**Conclusions:**

HF is a rare feature of NM, but it can negatively influence prognosis. Conventional HF therapy and/or heart transplant are the only reasonable strategy in these patients. Ivabradine is a useful, effective and safe drug for therapy in NM patients with HF and should be considered when resting HR remains high despite beta-blockers’ full titration or beta-blockers’ underdosing due to intolerance or side effects.

## Background

Nemaline myopathy (NM) is an uncommon congenital myopathy defined by the presence in muscle fibers of inclusions known as nemaline bodies (Greek nema = thread). In most cases muscle weakness and hypotonia are apparent from the neonatal period or infancy but fetal, childhood-onset and adult-onset forms are also recognized [1]. Cardiac involvement occurs in the minority of cases, although it can cause increasing weakness and physical disability [2]. No curative treatment is currently available for NM. We report a rare case of a patient with recently diagnosed adult-onset NM who developed progressive heart failure (HF) with reduced left ventricular ejection fraction (LVEF) in which optimal titration of medical therapy was hampered by symptomatic hypotension. The adjunction of ivabradine therapy had a decisive effect on the clinical course.

## Case presentation

**Figure 1:**
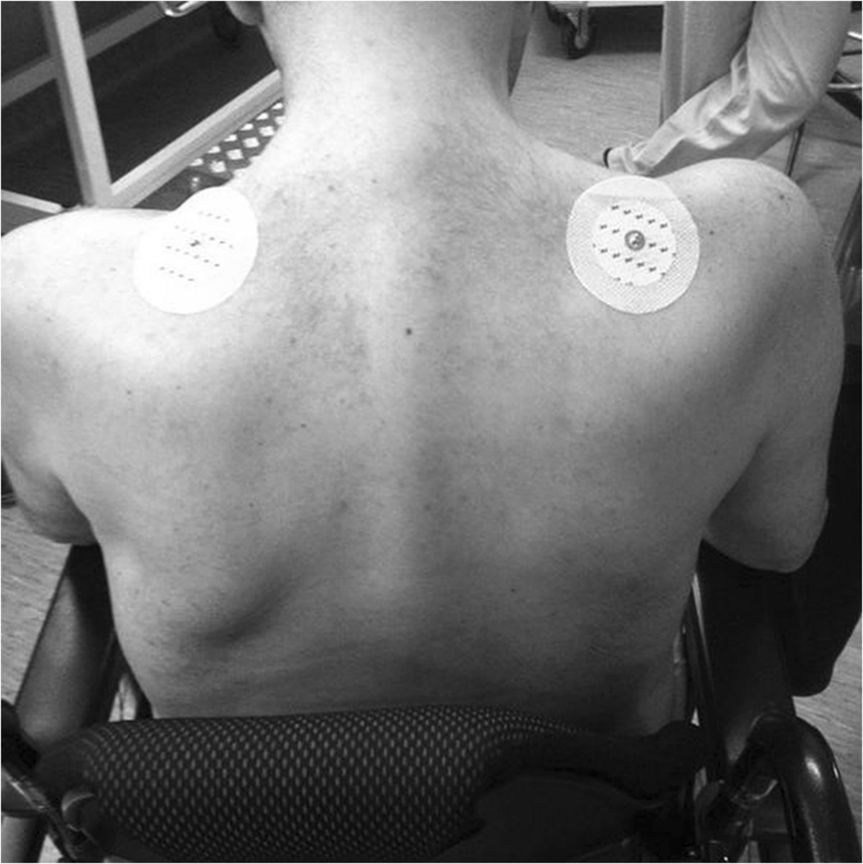
**Hypotonia of proximal upper extremities and shoulder girdles.**

The patient is a 37 year-old man. His medical history was characterized by surgery for frequent shoulder dislocations at the age of 24, a traumatic injury with rupture of anterior cruciate ligament in his left knee and chicken pox at the age of 30. He is a cigarette smoker and has familial history of arterial hypertension and dyslipidemia.In 2010 he started complaining of weakness in his upper limbs and shoulder girdles (Figure 1) and an abnormal posture with cervical hyperlordosis. In a few months he developed progressive hypostenia at upper extremities with involvement of lower extremities and marked inability to perform daily activities (like dressing, driving, climbing stairs). On September 2011 he was admitted to hospital for medical investigations.

At admission, resting heart rate (HR) and blood pressure were 92 beats per minute (bpm) and 100/70 mmHg, respectively. His body surface area (BSA) was 1.64 mq.

**Figure 2:**
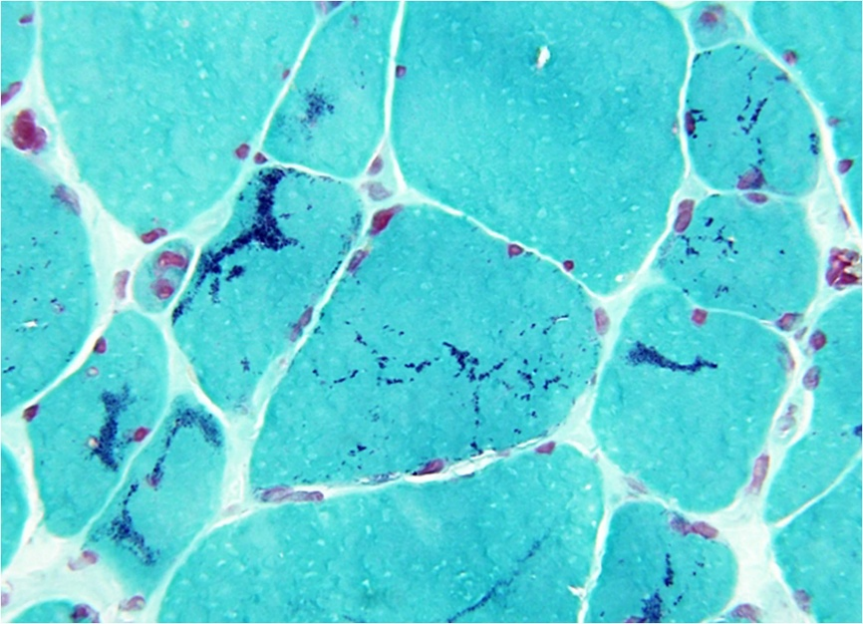
**Gomory trichrome staining showing Nemaline Rods.**

Thoracic and heart auscultation of the heart were unremarkable and no signs of pulmonary or systemic congestion were detected. Neurologic findings included severe weakness and hypotonia of upper extremities, mild weakness of lower extremities with slight reduction of deep tendon reflexes, mild weakness of the neck extensor muscles, inability to reach Mingazzini position (I and II) without help and waddling gait. No facial weakness nor ophthalmoplegia were detected.Electromyography highlighted bilateral myogenic suffering of the shoulder girdle with signs of secondary reorganizations of motor units. Muscle biopsy of biceps brachii showed marked variation in muscle fiber size, atrophic type I fiber predominance and marked disarray of contractile proteins and Z-disks; multiple cytoplasmic nemaline rods were visible in atrophic fibers with Gomori trichrome staining. Electron microscopy (Figure 2) revealed numerous sarcoplasmic inclusions with the typical ultrastructural features of nemaline bodies. Nemaline myopathy was diagnosed. No familiarity for NM was proven. The analysis of gene mutations is still in progress.

Electrocardiography (ECG) showed sinus rhythm, with a resting HR of 92 bpm and diffuse non-specific ST-T wave changes. Echocardiography showed left ventricular systolic dysfunction due to diffuse hypokinesia with a moderate reduction of left ventricular ejection fraction (LVEF = 42%), normal dimensions of cardiac chambers, moderate pericardial effusion with initial systolic collapse of the right atrium and no signs of pulmonary hypertension.

Spirometry revealed the presence of a moderate restrictive syndrome. He also underwent a cardiopulmonary test (VO_2_ max: 16.7 ml/kg/min) resulting in a moderate-severe reduction of functional capacity.

Blood test findings were normal except for a mild increase in liver function tests without organic evidence of liver abnormality at abdomen echography and computed tomography (CT) scan. Autoanticorpal screening, viral hepatitis and Human Immunodeficiency Virus (HIV) serology were negative. A monoclonal gammopathy of undetermined significance was found.

Pericardiocentesis was performed because of increasing pericardial effusion. No signs of infection or malignancy were noted on pericardial effusion on thoraco-abdominal CT scan.

The patient was treated with enalapril (2.5 mg b.i.d.), carvedilol (6.25 mg b.i.d.) and spironolactone (50 mg/die). Target HF doses of beta-blocker wasn't reached because of symptomatic hypotension. LVEF at discharge was 50%.

In the following months the patient started a program of intensive rehabilitation with a mild improvement in posture and gait although a worsening dysphagia was noted.

**Figure 3:**
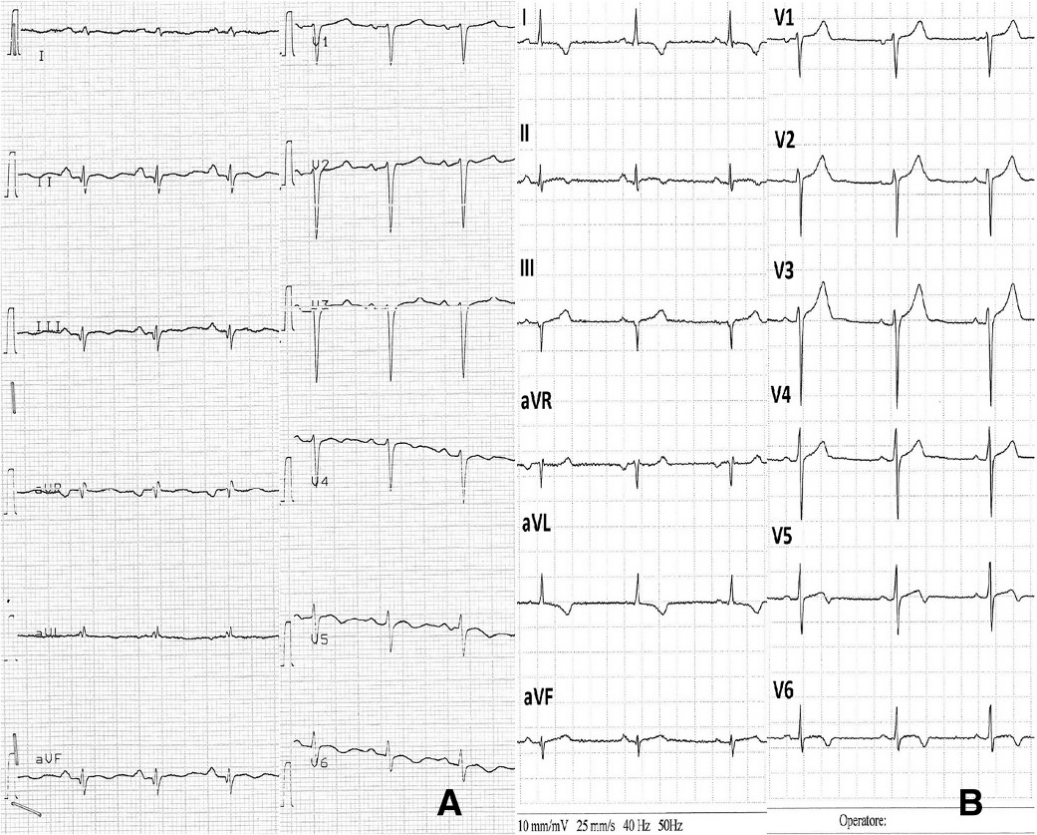
**A. ECG at baseline: sinus rhythm, 90 bpm; B. ECG at 6 month follow-up: sinus rhythm 55 bpm.**

**Figure 4:**
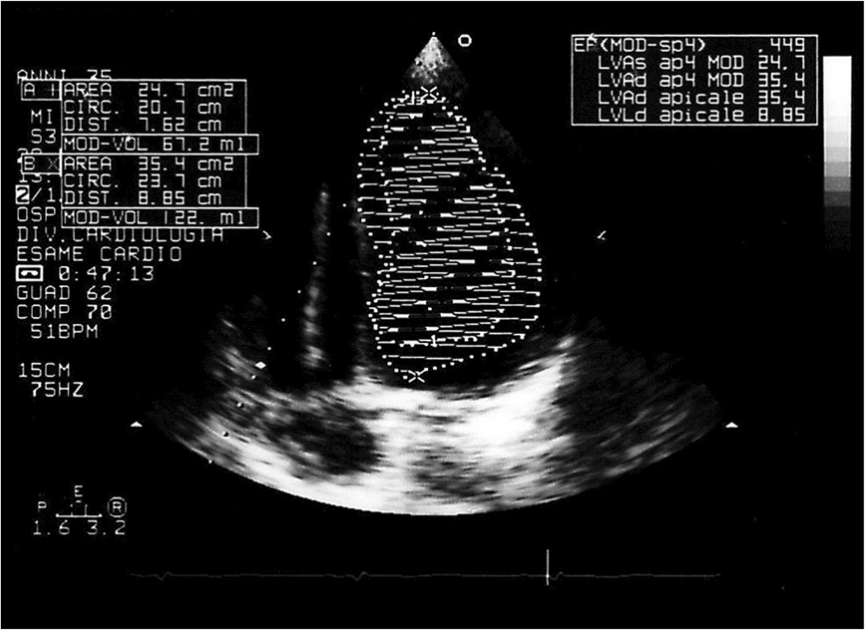
**Echocardiogram showing LVEF (45%) at 1 month follow up after ivabradine introduction.**

After an initial improvement of clinical conditions, increasing signs of systemic congestion occurred [progressive positive fluid balance, tender hepatomegaly, jugular distension, bilateral perimalleolar pitting edema, pleural effusion, New York Heart Association (NYHA) Class III-IV], together with dyspnea during speech, requiring admission to Intensive Care Unit (ICU). At admission, ECG showed sinus rhythm with resting HR 105 bpm; blood pressure was 90/60 mmHg. Echocardiogram revealed worsened biventricular systolic function [LVEF = 20%; Tricuspid Annular Plane Systolic Excursion (TAPSE) 13 mm], with signs of initial eccentric remodeling (End Diastolic Volume/BSA = 99 ml/mq) and mild pericardial effusion. Treatment with dobutamine and furosemide infusion improved hemodynamics but after few days three cardio-circulatory arrests occurred due to ventricular fibrillation and ventricular tachycardia requiring external defibrillation. An implantable cardioverter defibrillator (ICD) was then implanted for secondary prevention. Therapy was added with digossin, amiodarone and furosemide but patient's resting HR at discharge was still >70 bpm due to the aforementioned limitation in beta-blocker titration.On November 2011 a new rehabilitation program was started at our institution. Ivabradine (5 mg b.i.d.) was initiated with successful reduction of resting HR from 90 bpm to 55 bpm at 1 month follow up, also confirmed at sixth month follow up (Figure 3). Echocardiogram showed amelioration of left ventricular function at 3 month follow up (LVEF = 45%) (Figure 4). At 2,5 year follow up Echocardiogram revealed recovered biventricular function and significant reverse remodeling (LVEF = 55%; End Diastolic Volume = 41 ml/mq, TAPSE = 21 mm).

The patient is now able to carry on the rehabilitation program, achieving net improvement in neuromotor control of the limbs and trunk and less limitations in daily activities. No more hospitalizations for HF occurred since optimization of medical therapy with ivabradine was started with reduction of HR. However a single hospitalization occurred for aspiration pneumonia; because of persistent dysphagia, a tracheostomy tube was inserted to protect airways from further aspiration. At clinical follow up ivabradine was well tolerated, the compliance was satisfactory and no side effects occurred. The patient is regularly doing chest physiotherapy.

## Discussion

NM is a rare congenital myopathy characterized by muscle weakness, hypotonia and the presence in muscle fibers of inclusions known as nemaline bodies and a wide spectrum of phenotypes. NM is classified according to age of onset and severity of motor and respiratory involvement and includes the severe neonatal form, Amish NM, intermediate form, typical form, childhood-onset and adult-onset form [1]. In patients with adult-onset form of NM symptoms often develop between age 20 and 50 years without preceding symptoms or familial history [2]. No curative treatment is currently available for NM. A small number of affected patients have a monoclonal gammopathy in association with their myopathy which may be a marker of poor prognosis in individuals with adult-onset NM [3–5]. Cardiac involvement is uncommon and manifests as hyperthropic cardiomyopathy or, more frequently, as dilated cardiomyopathy with HF [6–9]. Notably, cardiac and respiratory involvement implies a worse prognosis [2]. Conventional HF therapy and/or heart transplant are, at the moment, the only reasonable strategies in these patients.

We reported a case of adult-onset NM with progressive HF. The clinical and pathological findings of our patient were similar to those of previously described cases of adult-onset nemaline myopathy [2–4, 10–12] including proximal muscle weakness, dysphagia, lack of familiarity involvement or preceding symptoms and monoclonal gammopathy. Symptomatic hypotension significantly limited optimal titration of Angiotensin Converting Enzyme (ACE)-inhibitors and mostly of beta-blockers with a consequent persistent elevated resting HR.

High resting HR has been shown to correlate with clinical outcome both in the general population and in patients with cardiovascular disease [13, 14]. Resting HR may also have an independent prognostic value in chronic HF and is a potentially modifiable cardiovascular risk factor [15–17]. Several HF clinical trials showed that reaching recommended target doses of beta-blockers results in better outcomes [18–21]. Moreover, recent meta-analysis of beta-blockers trials in HF found that the magnitude of HR reduction was more important than the achievement of target dose in predicting improved outcome [22, 23]. Notably, beta-blockers are generally well tolerated but side effects may occur which may require drug withdrawal or a dose reduction, as in the case of our patient [24].

High resting HR is currently considered a marker of decompensated HF and its persistence despite optimal beta-blocker titration or following beta-blocker undertitration (due to drug intolerance or side effects occurrence) constitutes an indication for treatment with ivabradine.

Ivabradine is a purely HR-lowering drug that acts by inhibiting the diastolic I_f_ current in the sinus node. It has proven to be effective and safe in improving clinical outcome in HF (mainly HF hospitalization and NYHA class) [25, 26]. It is noteworthy that ivabradine has no action on other channels in the heart or vascular system, then it does not affect myocardial contractility or atrio-ventricular conduction. A previous report by Taglia et al. [27] reported a similar case of adult-onset NM with cardiac involvement in whom ivabradine ameliorated the clinical picture. In this report beta-blocker was substituted with ivabradine after about 12 years of follow up, likely because of the severe respiratory insufficiency. Conversely instable hemodynamics and hypotension were predominant in our patient and requiring early therapeutic countermeasures to obtain a better hemodynamic compensation. Ivabradine introduction coincided with a significant clinical improvement, achieving resting HR <70 bpm and contractility improvement with increased LVEF. Also, no more hospitalizations for HF occurred since ivabradine was started and the patient was able to carry on the rehabilitation program with net improvement of his clinical conditions.

## Conclusions

HF is a rare feature of NM, but it can negatively influence prognosis. Conventional HF therapy and/or heart transplant are the only reasonable strategy in these patients. Ivabradine is a useful, effective and safe drug for therapy in NM patients with HF and should be considered when HR remains high even though ACE-inhibitors and beta-blockers are given or are underdosed due to intolerance or side effects.

## Consent

Written informed consent was obtained from the patient for publication of this case report and any accompanying images. A copy of the written consent is available for review by the Editor of this journal.

## Abbreviations

ACE: Angiotensin Converting Enzyme
b.i.d: bis in die
BSA: Body Surface Area
CT: Computed Tomography
EF: Ejection Fraction
HF: Heart Failure
HIV: Human Immunodeficiency Virus
HR: Heart Rate
ICD: Implantable Cardioverter Defibrillator
ICU: Intensive Care Unit
LVEF: Left ventricle ejection fraction
NM: Nemaline Myopathy
NYHA: New York Heart Association
TAPSE: Tricuspid Annular Plane Systolic Excursion
ECG: Electrocardiogram.
